# LiNbO_3_ and ZnO–Ni multilayer thin films as hybrid metamaterials towards tunable properties

**DOI:** 10.1039/d5ra08365f

**Published:** 2026-04-24

**Authors:** Nirali A. Bhatt, Lizabeth Quigley, Juanjuan Lu, Claire A. Mihalko, Aleem Siddiqui, Raktim Sarma, Haiyan Wang

**Affiliations:** a School of Materials Engineering, Purdue University West Lafayette IN 47907 USA; b School of Electrical and Computer Engineering, Purdue University West Lafayette IN 47907 USA hwang00@purdue.edu; c Sandia National Laboratories Albuquerque NM 87123 USA; d Center for Integrated Nanotechnologies, Sandia National Laboratories Albuquerque NM 87123 USA

## Abstract

Hybrid metamaterials (HMMs) are artificially designed materials made with two or more materials and present optical, magnetic, and mechanical responses that can be tailored. They have attracted great interest in various applications including advanced photonic and acoustic devices. HMMs have been demonstrated in the form of multilayer, particles in matrix, vertically aligned nanocomposites (VANs), and other morphologies. In this work, multilayer and VAN morphologies are integrated as complex HMMs by growing ZnO–Ni VAN and lithium niobate (LiNbO_3_, LNO) multilayer structures in either bi-layer stacks or multilayer stacks. The complex hybrid metamaterial system couples tailorable ferromagnetic and optical properties of ZnO–Ni VAN with ferroelectric LNO. The number of layers, interlayer selection, and thickness of each layer were varied to achieve tailorable physical properties and enhance coupling. The hyperbolic behavior can be tailored by varying the thickness of the ZnO–Ni layer. The magnetic anisotropy of the films, including coercivity and saturation magnetization, can be tuned by the number of film layers. Plus, the ferroelectric behavior can be tuned by the LNO layer thickness. This study demonstrates the growth of a new complex hybrid metamaterial system and its potential in plasmonics, integrated photonics, acoustic sensing, and data storage applications.

## Abstract 2

ZnO–Ni and LiNbO_3_ multilayer thin films were studied through varying layer number, layer thickness, and layer ordering to explore property tuning.

## Graphical abstract

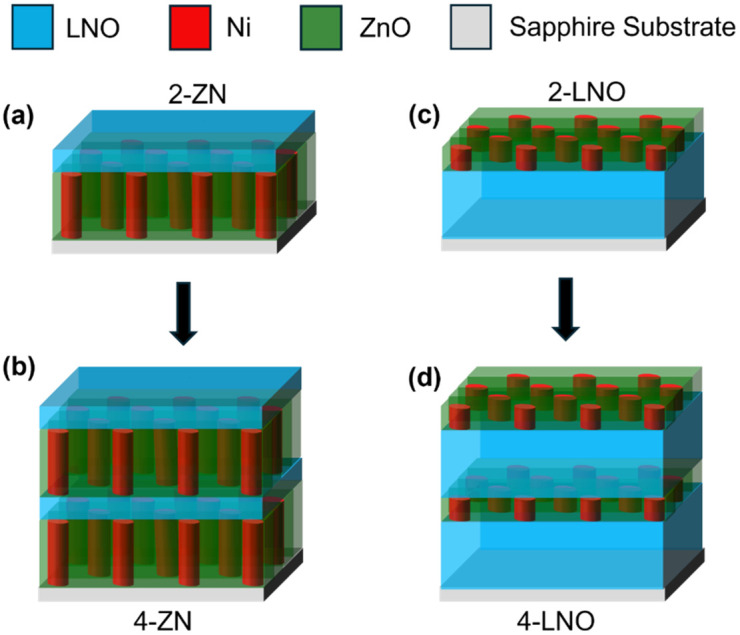

## 1 Introduction

Hybrid metamaterials (HMMs) are artificially designed materials made with two or more materials and present tailored optical, magnetic and mechanical responses.^1^ Because of their uniquely designed architectures and integration of different materials, novel properties have been demonstrated, including hyperbolic optical responses, negative refractive index in metamaterial antenna arrays,^2^ and self-responsive components of meta-lattices like in origami mechanical metamaterials.^3^ They have attracted great interest in various applications including advanced photonic and acoustic devices, and micro-mechanical systems (MEMS) devices. Most of the HMMs have been demonstrated in the form of multilayers, lithographically patterned nanostructures, and, recently, vertically aligned nanocomposites (VANs).

Multilayer thin films are made up of different material layers grown on top of each other. This creates a metamaterial that has a large amount of anisotropy resulting in the formation of unique properties. Varying the layer thickness and material selection plays a critical role in property tuning.^4^ Multilayer thin films can be made up of a mix of transparent conductive oxides,^5^ metals,^6^ ceramics (*e.g.*, oxides and nitrides),^7^ and, even polymers.^8^ A couple of advantages of multilayer thin films are the variety of methods that can be used to grow them and the property improvement/tuning/emergence that can occur. Both chemical and physical methods have been used to grow multilayer thin films.^9^ Plus, properties like improved resistivity,^10^ film strengthening,^11^ magnetic,^12^ and optical properties^13^ have been observed.

Lithographically patterned metamaterials are another major group of HMMs that include multilayer Ag and MgF_2_,^14^ and continuous Pd films,^15^ just to list a few. They have demonstrated low loss and other very novel optical and plasmonic properties. However their scale and resolution have been limited by the lithographic patterning methods, such as ∼40 nm by conventional wet lithographic patterning,^16^ 10 nm by e-beam lithography^17^ and 10 nm by focus ion beam.^18^ Recently the demonstration of VAN HMMs have shown the promises in making nanoscale HMMs with enhanced optical and magnetic anisotropy and tunability.^19^

In this work, we propose to integrate multilayer and VAN morphologies as complex hybrid metamaterials by growing ZnO–Ni VAN and lithium niobate (LiNbO_3_, LNO) multilayer structures in either bi-layer stack or multilayer stack fashion. Combining ZnO which is an n-type semiconductor with piezoelectric properties and Ni which is a ferromagnetic transition metal create a unique metamaterial system. In most ZnO–Ni thin films, Ni is the dopant which aids in tuning bandgap,^20^ improving transmittance,^21^ and surface roughness^22^ and the microstructure in these doped films varies from nanosheets,^23^ nanowires,^24^ and nanoparticles.^25^ There also have been studies that grow Ni as a secondary phase in ZnO–Ni thin films. Secondary phase growth of Ni has demonstrated property and nanostructure tuning based on deposition conditions which makes these metamaterial thin films good candidates for optoelectronic devices, sensors, and data storage applications.^19^ LNO on the other hand is a well-known ferroelectric with a trigonal crystal structure. This means that it can have either a hexagonal unit cell or a rhombohedral.^26^ LiNbO_3_ is the stoichiometric phase, but other phases can develop as well such as Li_3_NbO_4_ and LiNb_3_O_8_. Unlike LNO, the other phases are not ferroelectric. Though LNO is preferred, the methods to grow LNO are not very reproducible.

**Fig. 1:**
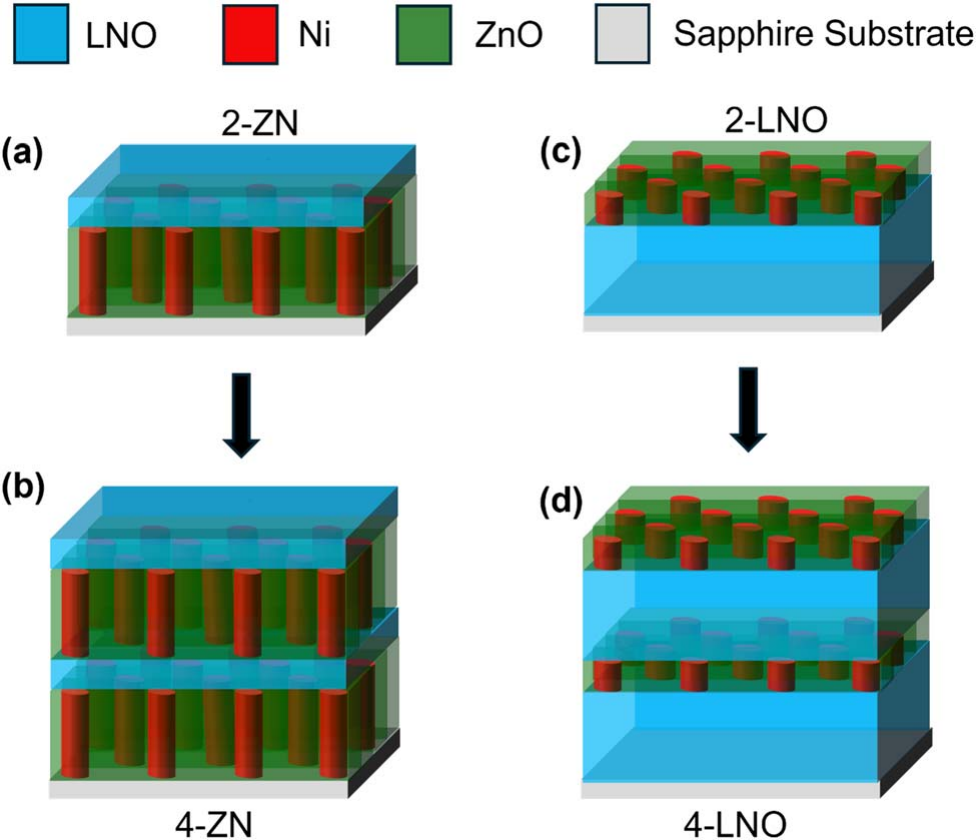
Schematic of the discussed thin films. The sample names have two parts. The number (either 2 or 4) is the number of layers in the multilayer thin films. Following the number, the abbreviation (either ZN (ZnO–Ni) or LNO (lithium niobate)) is the first layer grown on top the substrate. The first layer is always the thicker one and is followed by its counterpart. This can be repeated if the film has four layers. The samples are (a) 2-ZN, (b) 4-ZN, (c) 2-LNO, and (d) 4-LNO.

Specifically, we propose to explore the growth of LNO/ZnO–Ni multilayer thin films with varying layer number and thickness. The aim was to understand how the ordering of the LNO and ZnO–Ni would impact film properties. A schematic of the films proposed is shown in Fig. 1 and average layer thickness in Table 1. The error range for the thicknesses in Table 1 is ±1.3 nm. First, bilayer films of thick LNO and thin ZnO–Ni in addition to thick ZnO–Ni and thin LNO were grown. Then, the same two layers were grown on top to form a four layer film. The sample names have two parts. The number (either 2 or 4) is the number of layers in the multilayer thin films. Following the number, the abbreviation (either ZN (ZnO–Ni) or LNO (lithium niobate)) is the first layer grown on top the substrate. For example, sample 2-ZN means the sample has 2 layers with the ZnO–Ni being the first grown on top of the substrate and LNO grown on top of that. The abbreviation in the sample name is the first layer on grown on top of the substrate and will always be thicker. Thin film characterizations including microstructure, optical, magnetic, and ferroelectric properties were conducted to explore the potential for tailored physical properties in the complex HMMs. This tunable complex HMMs system could demonstrate potentials for composite multiferroic applications and acoustic wave devices.

**Table 1:** A table of the average thicknesses of the different layers in the samples discussed

Film	Avg. ZnO–Ni layer thickness	Avg. LNO layer thickness
2-ZN	65 nm	35 nm
4-ZN	75 nm	50 nm
2-LNO	35 nm	145 nm
4-LNO	20 nm	110 nm

## 2 Materials and methods

### 2.1 Thin film growth

The multilayer thin films discussed in this paper were grown using laser-molecular beam epitaxy (laser-MBE) (vacuum at least 10^−8^ torr or better). All films were deposited on c-cut sapphire substrates with a KrF excimer laser (*λ* = 248 nm) using a ZnO(70 mol%)–Ni(30 mol%) composite target and a pure LNO target. For film 2-ZN, the ZnO–Ni layer was grown with 3000 pulses, 5 Hz laser frequency, 620 °C, 420 mJ laser energy, and vacuum deposition. The pure LNO was grown under the same conditions but with 1000 pulses instead. This was repeated for the 4-ZN sample. For samples 2-LNO and 4-LNO, the pure LNO was grown with 3000 pulses, 5 Hz laser frequency, 620 °C, 420 mJ laser energy, and vacuum deposition. The ZnO–Ni was grown under the same conditions but with 1000 pulses instead. For the electrical measurements, an aluminum doped ZnO (AZO) was deposited under the films as the bottom electrode (1250 pulses, 5 Hz, 780 °C, 50 mTorr oxygen partial pressure during deposition, 450 mJ laser energy, and 100 torr oxygen pressure at 500 °C for 30 minutes annealing).

### 2.2 Microstructure characterization

The microstructure characterization in this paper was performed using X-ray diffraction (XRD), transmission electron microscopy (TEM), scanning transmission electron microscopy (STEM), and energy-dispersive X-ray spectroscopy (EDS). A Malvern PANalytical Empyrean X-ray diffractometer (XRD) from Worcestershire, UK was used to conduct q–2q scans with Cu Kα (*l* = 0.154 nm) radiation. The ThermoFisher TALOS F200X TEM was utilized to get the TEM/STEM/EDS-mapping images. The cross-section TEM samples were prepared by a standard sample preparation procedure consisting of manual grinding, dimple polishing, and ion milling (PIPS 695 system, 5 keV).

### 2.3 Optical measurements

Optical measurements were obtained using a J. A. Woollam RC2 spectroscopic ellipsometer for ellipsometry measurements. The ellipsometric parameters psi (*Ψ*) and delta (*Δ*) were measured at incident angles from 55° to 75° in 10° increments over a range of 210–2500 nm. The dielectric permittivity was then modeled using appropriate oscillators.

### 2.4 Magnetic measurements

Magnetic properties were conducted using the MPMS-3 EverCool SQUID magnetometer in the user facility of the Birck Nanotechnology Center at Purdue University, see birck.research.purdue.edu (https://birck.research.purdue.edu). Both the parallel and perpendicular directions were tested with the applied magnetic field and the scans were done at temperatures of 10 K and 300 K.

### 2.5 Ferroelectric measurements

Ferroelectric property measurements were conducted using the Radiant Technologies Precision LC II Ferroelectric tester.

## 3 Results

### 3.1 Microstructural analysis

**Fig. 2:**
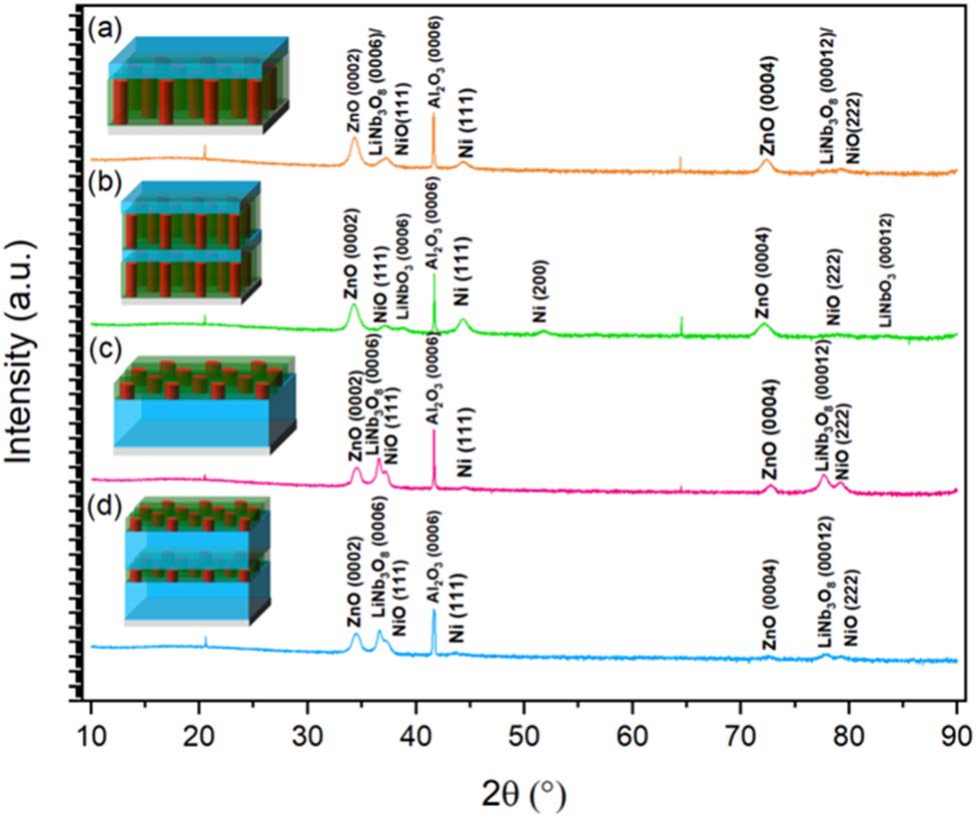
XRD *θ*–2*θ* plot of sample (a) 2-ZN, (b) 4-ZN, (c) 2-LNO, and (d) 4-LNO.

X-Ray diffraction (XRD) *θ*–2*θ* measurements were conducted to understand the crystallographic structure and out-of-plane texturing property of the multilayered films as seen in Fig. 2. First, 2-ZN, 2-LNO, and 4-LNO (Fig. 2a, c and d, respectively) demonstrate an out-of-plane texture relation of ZnO(0002) ∥Ni(111)∥ LiNb_3_O_8_(0006) on c-cut Al_2_O_3_(0006). 4-ZN, as seen in Fig. 2b, shows an out-of-plane textured growth of ZnO, Ni, LNO, but there is also the growth of Ni(200). This additional Ni(200) orientation could be from the ZnO–Ni layer deposited on top of the first LNO layer. To minimize and accommodate strain at the upper interface of the LNO layer with the ZnO–Ni layer, adatom nucleation may have favored the nucleation of a mix of Ni(111) and Ni(200).

Additionally, the 2-LNO and 4-LNO films have very low intensity Ni(111) peaks while the 2-ZN and 4-ZN films have relatively higher intensity Ni(111) peaks. This difference can be attributed to what material is underneath the ZnO–Ni layer. For the ZN films, the hexagonal sapphire substrate is underneath the ZnO–Ni layer which has a good lattice match to ZnO. In contrast, for the ZnO–Ni is grown on top of the LNO layer. This likely causes the Ni to grow as NiO, resulting in the minor Ni(111) peak. The formation of the LiNb_3_O_8_ phase is likely related to the high volatility of Li during deposition.^27^

**Fig. 3:**
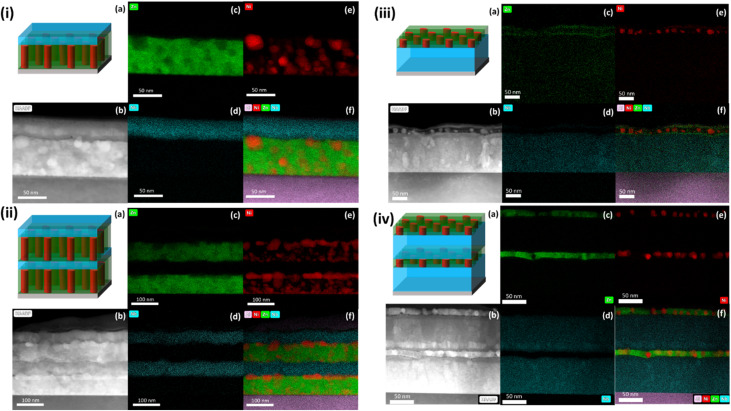
Microstructure characterization of the samples. (i(a)) Schematic of sample 2-ZN, (i(b)) cross-section STEM and (i(c)) EDS mapping of Zn, (i(d)) Nb, (i(e)) Ni, and (i(f)) combined Al, Ni, Zn, Nb. (ii(a)) Schematic of sample 4-ZN, (ii(b)) cross-section STEM and (ii(c)) EDS mapping of Zn, (ii(d)) Nb, (ii(e)) Ni, and (ii(f)) combined Al, Ni, Zn, Nb. (iii(a)) Schematic of sample 2-LNO, (iii(b)) cross-section STEM and (iii(c)) EDS mapping of Zn, (iii(d)) Nb, (iii(e)) Ni, and (iii(f)) combined Al, Ni, Zn, Nb. (iv(a)) Schematic of sample 4-LNO, (iv(b)) cross-section STEM and (iv(c)) EDS mapping of Zn, (iv(d)) Nb, (iv(e)) Ni, and (iv(f)) combined Al, Ni, Zn, Nb.

The growth morphology of the multilayer thin films with different layer thicknesses was characterized using STEM and EDS. The images of sample 2-ZN are shown in Fig. 3i(a–f). The ZnO–Ni layer is ∼65 nm and the LNO layer is ∼35 nm. ZnO is the matrix material while Ni is the secondary phase. Ni grows as nanoparticles with a thin layer of Ni nucleation seen at the substrate-ZnO–Ni interface. The interface between the LNO and ZnO–Ni layer does not show Ni diffusion into the LNO layer.

Sample 4-ZN is shown in Fig. 3ii(a–f). The ZnO–Ni layers are ∼75 nm while the LNO layers are around ∼50 nm. There is a clear interface between the layers with no diffusion of the Ni into the LNO layer. Additionally, there is a large amount of Ni in the ZnO–Ni layer that is highly segregated at the top of the layer as horizontally elongated nanostructure. The remainder of Ni not at the top of the layers grows as nanoparticles, with some areas with the nanoparticles stacked on top of one another. Plus, the Ni nanoinclusions on the topmost layer look more random with a range of particle shapes and sizes as compared to the Ni present in the layer grown directly on top of the substrate. A prior modeling study discussing metal and carbon film growth reported that Ni segregation near the top is a result of the different surface tension between the ZnO and Ni and the low miscibility of the phases. Additionally, coalescence of Ni increases as the Ni content increases.^28^ This may also be due to the additional time necessary to deposit the extra material necessary for multilayer films. The increased time at the raised deposition temperature for the Ni inclusions to minimize their total surface energy by coalescing into larger inclusions.^29^ This, combined with the effects of low miscibility and high surface energy cause the Ni inclusions to not only concentrate at the surface but to likely coalesce into a larger mass.^28–30^ This phenomenon is not nearly as evident in the upper layer due to the lower time and differing material environment.

Fig. 3iii(a–f) shows the STEM and EDS images for sample 2-LNO. The LNO layer is ∼145 nm and the ZnO–Ni layer is ∼35 nm. In contrast to sample 2-ZN, the growth of Ni in 2-LNO is much different. The Ni grows as evenly spaced nanoparticles on top of the LNO layer. The layer thickness of ZnO–Ni is a factor as well as the material the ZnO–Ni is grown on. Unlike 2-ZN, the ZnO–Ni in 2-LNO is deposited on top of the LNO which has different surface energy and lattice parameters. Consequently, Ni needs to find the right seeding sites for nucleation that minimize its surface energy. The nanoparticle growth could be due to the minimization of the surface energy of Ni on LNO. Conversely, in 2-ZN, the Ni forms a thin layer at the substrate–layer interface. This is consistent with the behavior observed in the ZN samples and suggests that the Ni/Al_2_O_3_ interface has a lower interfacial energy than that of Ni/LNO.

Fig. 3iv(a–f) shows STEM and EDS images of sample 4-LNO. The LNO layers are ∼110 nm while the ZnO–Ni layers are ∼20 nm. Similar to the 2-LNO case, the Ni grows as nanoparticles and there is a clear interface between all the layers with no visible diffusion between the layers.

### 3.2 Optical property tuning

**Fig. 4:**
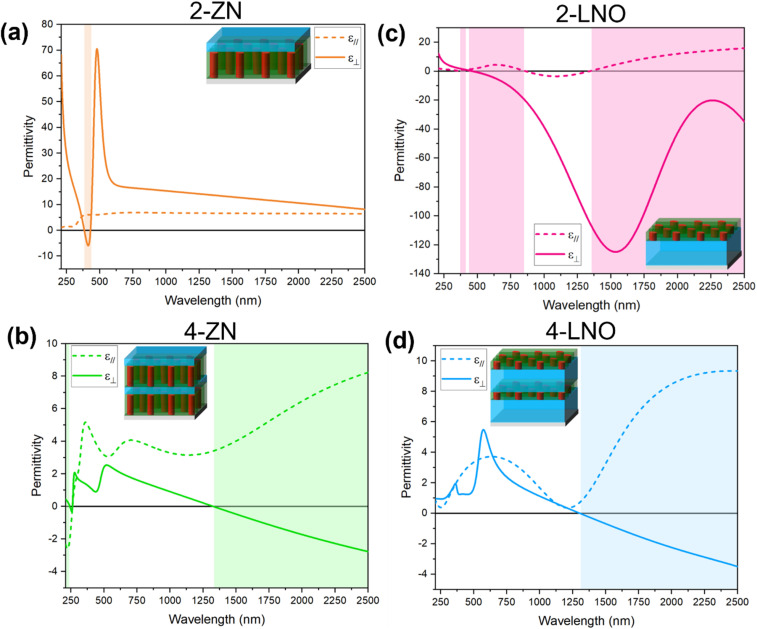
Optical permittivity of (a) 2-ZN, (b) 4-ZN, (c) 2-LNO, (d) 4-LNO. The highlighted regions represent wavelengths where hyperbolic behavior is present.

Considering the potential anisotropic optical properties of the multilayer thin films and the presence of the Ni phase in dielectrics, optical characterization was conducted. Specifically, ellipsometry measurements were conducted to understand the in-plane and out-of-plane permittivity to evaluate potential hyperbolic behavior. Hyperbolic behavior is present if a material behaves as a dielectric in one direction and as a metal in another for a range of wavelengths. There are two types of hyperbolic behavior: Type-I and Type-II. Type-I is when the *ε*_∥_ > 0 and *ε*_⊥_ < 0 while Type-II is *ε*_∥_ < 0 and *ε*_⊥_ > 0. All films demonstrate hyperbolic behavior as seen from Fig. 4 though the wavelength ranges differ for each sample. Fig. 4a shows the optical permittivity for sample 2-ZN. The hyperbolic dispersion is in the range of 384–430 nm with negative out-of-plane permittivity (*ε*_⊥_) and positive in-plane permittivity (*ε*_∥_) making it Type-I. Sample 4-ZN is seen in Fig. 4b. There are two wavelength ranges where hyperbolic dispersion is seen for this sample: 210–242 nm where *ε*_∥_ < 0 and *ε*_⊥_ > 0 (Type-II) and 1327–2500 nm with *ε*_∥_ > 0 and *ε*_⊥_ < 0 (Type-I). Fig. 4c shows permittivity for sample 2-LNO. There are three hyperbolic dispersion ranges seen: 361–371 nm (*ε*_∥_ < 0 and *ε*_⊥_ > 0; Type-II), 462–862 nm (*ε*_∥_ > 0 and *ε*_⊥_ < 0; Type-I), and 1341–2500 nm (*ε*_∥_ > 0 and *ε*_⊥_ < 0; Type-I). Sample 4-LNO is seen in Fig. 4d. 4-LNO demonstrates hyperbolic behavior in the wavelength range of 1303–2500 nm (*ε*_∥_ > 0 and *ε*_⊥_ < 0; Type-I).

Especially for the 2-LNO and 4-ZN samples, there is a transition from Type-II hyperbolic to Type-I. The tuning of the dispersion wavelengths is due to the differences in permittivity of the two different layer materials and the volume fraction of metal and dielectric.^31^ This is interesting because Type-I hyperbolic behavior is seen in nanowire/vertically aligned nanocomposite (VAN) structure while Type-II is typically seen in layered films. Clearly, depositing a nanocomposite thin film with a layered dielectric and ZnO–metal structure results in the development of a Type-I multilayered film.

Having VAN in a multilayer structure creates a highly anisotropic thin film. Additionally, these films have varying metallic nanostructures. This complex structure introduces strain tuning which is jointly influenced by the multilayer and VAN which then varies the hyperbolic dispersion in the films. VAN, unlike layered growth, has both in-plane and out-of-plane strain. The in-plane strain arises at the film–substrate interface and between different film layers. The out-of-plane strain arises at the interface between the matrix and nanopillar in the VAN. Consequently, especially for the 4 layered films which have different layers atop another, the in-plane strain plays a crucial role in permittivity. Having different dielectric layers of varying thicknesses has shown to impact VAN pillar diameter and metal phase shape.^32^ Since VAN usually has long out-of-plane metal pillars, the out-of-plane permittivity is negative as that is the property of metals. Oxides/dielectrics on the other hand have positive permittivity.

Additionally, there is tuning of the epsilon near zero (ENZ) values. At ENZ, there can be large enhancements in the light-matter interactions, which can be very advantageous for linear and nonlinear optical phenomenon such as large refractive index tuning and enhanced harmonic generation.^33^ Film 2-ZN has out-of-plane ENZ at 384 nm and 430 nm. Film 4-ZN has in-plane ENZ at 262 nm and out-of-plane ENZ at 261 nm and 1327 nm. Film 2-LNO has in-plane ENZ at four points (361 nm, 372 nm, 862 nm, 1341 nm) and one out-of-plane ENZ at 462 nm. Lastly, 4-LNO has one out-of-plane ENZ value at 1303 nm.

### 3.3 Magnetic property tuning

**Fig. 5:**
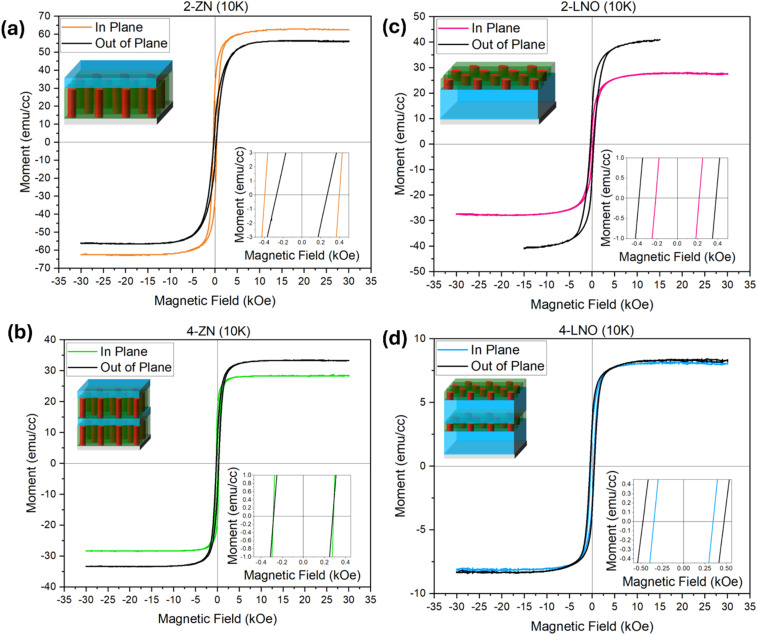
Magnetic data of magnetic moment *versus* magnetic field for (a) 2-ZN, (b) 4-ZN, (c) 2-LNO, and (d) 4-LNO measured at 10 K. The inset graph seen in all plots is to show the coercivities.

The multilayer films in this study also demonstrate magnetic properties due to the ferromagnetic Ni. Not only is Ni ferromagnetic, but it also varies its morphologies in the samples. This makes the magnetic property tuning in these multilayer thin films possible. Fig. 5 shows the magnetic hysteresis loops for the samples at 10 K and corresponding M–H loops. It is apparent that all samples are soft magnetic materials defined by the narrow hysteresis behavior. Soft magnetic materials like Ni have high saturation magnetization, magnetic susceptibility, and low coercivity and remanent magnetization.^34,35^ This makes these films easily magnetized and attracted to other magnetic materials. First, comparing together the 2-ZN (Fig. 5a) and 4-ZN (Fig. 5b) films with their counterparts of 2-LNO (Fig. 5c) and 4-LNO (Fig. 5d), there is a commonality in the decrease in the saturation magnetization as the number of film layers increases. This can be due to the decrease in stress and the larger role that surface energy plays.^36^ Clearly, the total thickness of the films plays a role in the saturation magnetization. The microstructure clearly influences the magnetic properties. Film 2-ZN demonstrates a larger in-plane coercivity than out-of-plane while film 4-ZN has both in-plane and out-of-plane the same. Coercivity is impacted by grain size and stress. Therefore, as the number of layers increases, the stress in the film decreases. Previous work has shown that stress can result in the magnetostrictive effect.^36^ As the number of film layers increases, the film thickness becomes larger causing a reduction in stress and therefore the coercivity. Additionally, coercivity is dependent on grain size. From the XRD results in Fig. 2a and b for film 2-ZN and 4-ZN, there is a Ni(200) peak that forms as the number of layers increases. This additional texture can be present in the grain therefore causing the 4-ZN sample to have coercivity similar to that of the in-plane 2-ZN even though the number of layers is more. When it comes to the 4-LNO sample, both the in-plane and out-of-plane hysteresis loops look stacked atop one of another. This likely means that the thicker LNO layer and smaller concentration of Ni result in lower anisotropy. As a general observation, all in-plane and out-of-plane coercivities are comparable in each sample. The presence of NiO, a antiferromagnetic material, had an influence on the coercivity by making it very low.

The magnetic data for the samples measured at 300 K is shown in Fig. S1. The coercivity for all samples is small as can be seen from the inset graphs.

### 3.4 Ferroelectric property tuning

**Fig. 6:**
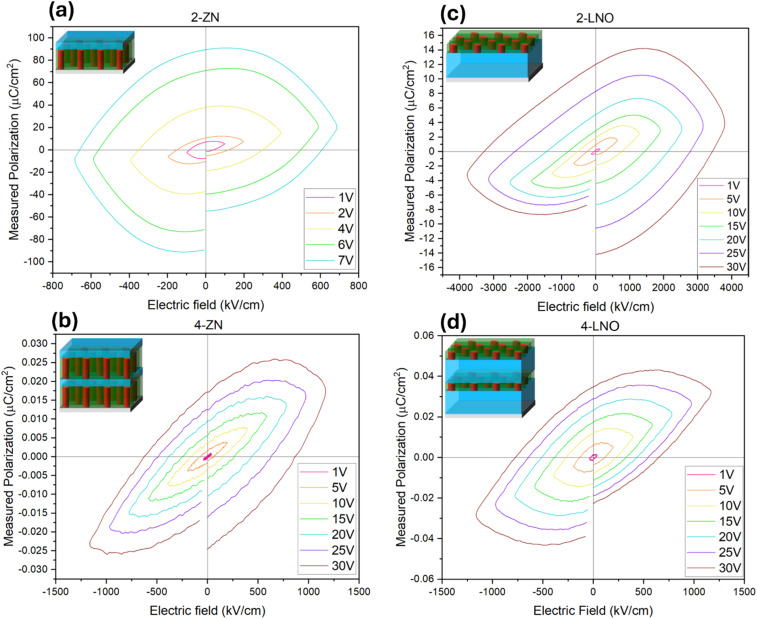
Ferroelectric polarization *versus* electric field (*P*–*E*) loops for (a) 2-ZN, (b) 4-ZN, (c) 2-LNO, and (d) 4-LNO. Different applied voltages are measured.

Ferroelectric measurements were conducted by ferroelectric tester a probe station where voltage was applied to the samples and the resulting polarization (*P*) was measured. The sample thickness was considered for each sample and is represented by the electric field (*E*) in Fig. 6 in the *P*–*E* loop. Similar to the ferromagnetic hysteresis loop, a proper ferroelectric hysteresis loop shows the coercive field, remanent polarization, and saturation polarization. Tabulated values for the coercive field and saturation polarization for each sample are available in Tables S1–S4 in SI. The shape of the hysteresis loop reveals the type of ferroelectric material. From the XRD data seen previously, LNO is a ferroelectric material in this multilayer system, but majority of the growth was LiNb_3_O_8_(0006) instead of the ferroelectric LNO. The measured *P*–*E* loops present some of the characteristics of hysteretic properties but with some leakage currents.^37^ The shape of *P*–*E* loop for the 2-ZN and 2-LNO samples seen in Fig. 6a and c respectively are predominantly ‘football’ shaped and as the layer count increases in 4-ZN (Fig. 6b) and 4-LNO (Fig. 6d), the loop begins to have a similar form of a typical ferroelectric hysteresis property. Overall, the ferroelectric polarization behavior in all films seen in Fig. 6 is due to leakage rather than true ferroelectric switching. There is clear dielectric loss resulting from the phases in the films. Even other studies that measured the ferroelectric hysteresis of LNO, show a high leakage hysteresis similar in shape to what is seen in Fig. 6b and d.^38^ This leakage is attributed to oxygen vacancies, leakage pathways, present on the surface of the film. With this benchmark^38^ however, the saturation polarization is slightly bigger than the 30 V loop in Fig. 6d while the coercive field is closest to the 1 V loop in Fig. 6d.

## 4 Discussion

The ZnO–Ni/LNO multilayer thin films integrate multiple functionalities and demonstrate property tunability all in one system. This ZnO–Ni/LNO multilayer system demonstrates hyperbolic dispersion tuning, specifically with wavelength ranges where *ε*_∥_ > 0 and *ε*_⊥_ < 0 (Type-I). This hyperbolic dispersion can be used in applications such as for negative refraction and hyperlenses. However, such dispersion is typically difficult to achieve in layered thin films especially in the near infrared (750 nm to 2500 nm)^39^ and visible regions (380 nm to 750 nm) of the electromagnetic spectrum.^40^ This means that combining VAN with the multilayer design could be an effective approach for optical tuning and achieving hyperbolic dispersion in the near infrared and visible range. Similarly, TiN- and TaN–CoFe_2_ multilayer films demonstrated this ability previously.^41^ Interestingly, the hyperbolic dispersion changed from Type-II to Type-I as the number of layers increased as well as the Type-I behavior occurring in the near infrared region. Overall, the tunable hyperbolic dispersion and ENZ properties can be utilized in radio-photonic devices such as dynamic reconfigurable metasurface antennas. These antennas provide lower power usage due to the metamaterial design.^42^ Other metamaterial applications include plasmonic waveguides,^43–45^ multilayered metamaterial structures for information transfer,^46^ controllable Weyl nodal line semimetals,^47^ and near-field routing of hyperbolic polaritons.^48^

One limitation of the previous reports is the lack of stoichiometric LNO growth. A prior study by Paldi *et al.*^49^ achieved stoichiometric growth of LNO by using a Li-rich target so that excess Li in the target could compensate the Li loss during the growth. This is a method that can be implemented in future work. Interestingly though, sample 4-ZN did show a minor stoichiometric LNO XRD peak in Fig. 2, which was not present in sample 2-ZN. This suggests that multilayer growth could be another method to achieve stoichiometric LNO.

Considering the excellent acoustic properties of LNO, such as high piezoelectricity and electromechanical coupling coefficient, LNO is ideal for surface acoustic wave devices. Coupling magnetic nanostructures with LNO could lead to magnetoelectric coupling in the composite form which can be used for magnetic field sensing and exploring magnon–phonon coupling such as nonreciprocal devices. Further property analyses such as magneto–electric coupling measurements on these multilayer LNO/ZnO–Ni nanocomposite structures could be conducted for optimized multilayer structures for enhanced coupling. Selection of magnon-based magnetic nanoinclusions, such as Y_3_Fe_5_O_12_ (YIG) and Ni_80_Fe_20_ in the multilayer designs could lead to novel magnon–phonon coupling materials for future LNO-based nonreciprocal devices.

## 5 Conclusion

The ZnO–Ni/LNO multilayer system was deposited with different 2-layer and 4-layer designs to integrate HMM and ferromagnetic ZnO–Ni nanostructures with LNO layers. Their optical, magnetic and ferroelectric properties were characterized and compared. They present multiple new features: (1) the formation of stoichiometric LNO layers in 4-ZN samples; (2) the growth of various Ni microstructures not previously seen in ZnO–Ni thin films *e.g.*, Ni nanoparticle growth; (3) the presence of obvious hyperbolic behavior, both Type-I and Type-II, and the ability to tune the ENZ point. The findings suggest that the incorporation of the ZnO–Ni system is an effective approach to make the out-of-plane permittivity negative in multilayer growth; and, (4) tuning of ferromagnetic and ferroelectric behavior. Despite the fact that the stoichiometric LNO growth is to be further improved, this ZnO–Ni/LNO system could show promise in future photonic and acoustic devices.

## Author contributions

Conceptualization, investigation, methodology, writing – original draft, writing – review and editing, N. A. B.; investigation, writing – review and editing, L. Q., J. L., and C. M.; funding acquisition, supervision, writing – review and editing, R. S. and A. S.; conceptualization, funding acquisition, supervision, writing – review and editing, H. W. All authors have read and agreed to the published version of the manuscript.

## Conflicts of interest

There are no conflicts of interest to declare.

## Supplementary Material

RA-016-D5RA08365F-s001

## Data Availability

The data supporting this article have been included as part of the supplementary information (SI). Supplementary information: the plots of magnetic properties (M–H loops) under 300 K of all the samples and the table summary of the key magnetic properties. See DOI: https://doi.org/10.1039/d5ra08365f.
