# Supplementary material for: LiNbO3 and ZnO–Ni multilayer thin films as hybrid metamaterials towards tunable properties
Source: RSC Adv. 2026 Apr 24;16(24):21781–90. doi: 10.1039/d5ra08365f (PMC13108577; doi:10.1039/d5ra08365f)
Supplement: RA-016-D5RA08365F-s001 [file RA-016-D5RA08365F-s001.pdf]

Supplementary Information (SI) for RSC Advances.
This journal is © The Royal Society of Chemistry 2026

Supplementary Information

$LiNbO_3$ and ZnO-Ni Multilayer Thin Films as Hybrid Metamaterials Towards Tunable Properties

Authors: Nirali A. Bhatt[1], Lizabeth Quigley[1], Juanjuan Lu[1], Claire Mihalko[1], Aleem Siddiqui[3], Raktim Sarma[3,4], Haiyan Wang[1,2,*]

[1] School of Materials Engineering, Purdue University, West Lafayette, IN 47907, USA

[2] School of Electrical and Computer Engineering, Purdue University, West Lafayette, IN 47907, USA

[3] Sandia National Laboratories, Albuquerque, NM 87123, USA

[4] Center for Integrated Nanotechnologies, Sandia National Laboratories, Albuquerque, NM 87123, USA

[*]Author to whom correspondence should be addressed: hwang00@purdue.edu

Supporting Information

The supporting information includes the following.

Figure S1: Magnetic data of magnetic moment versus magnetic field for (a)2-ZN, (b)4-ZN, (c)2-LNO, and (d)4-LNO measured at 300K. The inset graph seen in all plots is to show the coercivities.

Table S1: Extracted saturation polarization ($\mathbf{P_{Max}}$) and coercive Field ($\mathbf{E_C}$) from ferroelectric polarization versus electric field (P-E) loops for sample 2-ZN.

Table S2: Extracted saturation polarization ($\mathbf{P_{Max}}$) and coercive Field ($\mathbf{E_C}$) from ferroelectric polarization versus electric field (P-E) loops for sample 4-ZN.

Table S3: Extracted saturation polarization ($\mathbf{P_{Max}}$) and coercive Field ($\mathbf{E_C}$) from ferroelectric polarization versus electric field (P-E) loops for sample 2-LNO.

Table S4: Extracted saturation polarization ($\mathbf{P_{Max}}$) and coercive Field ($\mathbf{E_C}$) from ferroelectric polarization versus electric field (P-E) loops for sample 4-LNO.

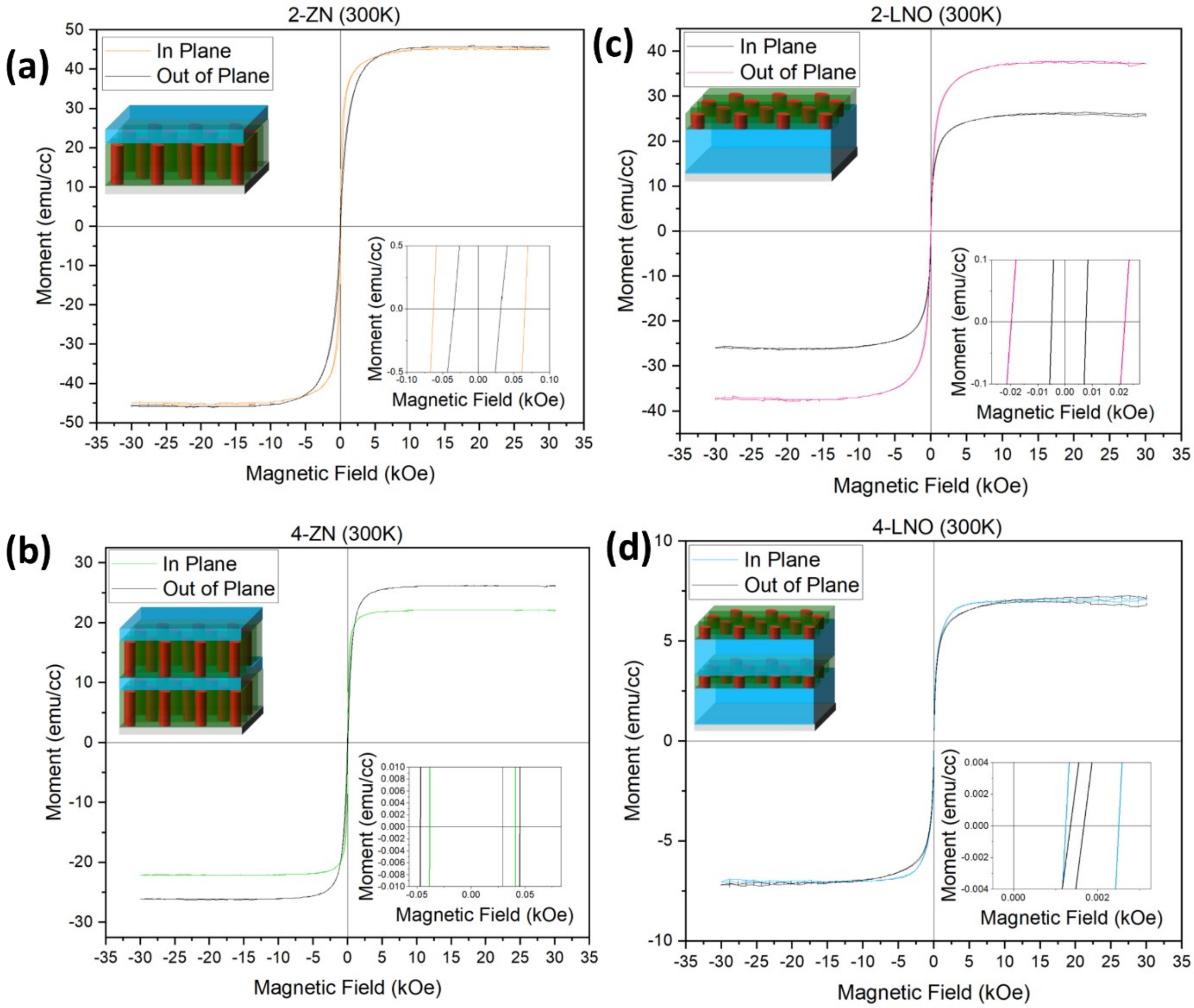

Figure S1: Magnetic data of magnetic moment versus magnetic field for (a)2-ZN, (b)4-ZN, (c)2-LNO, and (d)4-LNO measured at 300K. The inset graph seen in all plots is to show the coercivities.

Table S1: Extracted saturation polarization (**P_{Max}**) and coercive Field (**E_C**) from ferroelectric polarization versus electric field (P-E) loops for sample 2-ZN.

**2-ZN**							
	**1V**	**2V**	**3V**	**4V**	**5V**	**6V**	**7V**
**P_{Max} (μC/cm^2)**	7.76	12.33	24.75	39.28	60.57	72.94	91.09
**E_C (kV/cm)**	61.87	134.77	240.59	324.23	408.64	528.60	633.42

Table S2: Extracted saturation polarization (**P_{Max}**) and coercive Field (**E_C**) from ferroelectric polarization versus electric field (P-E) loops for sample 4-ZN.

**4-ZN**							
	**1V**	**5V**	**10V**	**15V**	**20V**	**25V**	**30V**
**P_{Max} (μC/cm^2)**	0.0011	0.0047	0.0079	0.013	0.016	0.020	0.026
**E_C (kV/cm)**	8.48	62.88	157.67	255.00	411.35	557.31	746.06

Table S3: Extracted saturation polarization (**P_{Max}**) and coercive Field (**E_C**) from ferroelectric polarization versus electric field (P-E) loops for sample 2-LNO.

**2-LNO**							
	**1V**	**5V**	**10V**	**15V**	**20V**	**25V**	**30V**
**P_{Max} (μC/cm^2)**	0.39	1.92	3.57	5.03	7.32	10.54	14.21
**E_C (kV/cm)**	17.50	107.59	278.01	482.40	764.87	1104.08	1435.82

Table S4: Extracted saturation polarization (**P_{Max}**) and coercive Field (**E_C**) from ferroelectric polarization versus electric field (P-E) loops for sample 4-LNO.

**4-LNO**							
	**1V**	**5V**	**10V**	**15V**	**20V**	**25V**	**30V**
**PMax (μC/cm2)**	0.0017	0.0069	0.015	0.020	0.027	0.034	0.040
**E_C (kV/cm)**	20.63	121.95	240.63	345.37	462.03	569.82	693.97
